# Comparison of morbidity and mortality of very low birth weight infants in a Central Hospital in Johannesburg between 2006/2007 and 2013

**DOI:** 10.1186/s12887-015-0337-4

**Published:** 2015-03-13

**Authors:** Daynia E Ballot, Tobias Chirwa, Tanusha Ramdin, Lea Chirwa, Irma Mare, Victor A Davies, Peter A Cooper

**Affiliations:** Departments of Paediatrics and Child Health, University of the Witwatersrand, Johannesburg, South Africa; Department of Public Health, University of the Witwatersrand, Johannesburg, South Africa; Department of Surgery, Faculty of Health Sciences, University of the Witwatersrand, Private Bag 3, Wits, 2050 Johannesburg, South Africa

**Keywords:** Infant, Very low birth weight, Premature, Neonatal mortality

## Abstract

**Background:**

Health protocols need to be guided by current data on survival and benefits of interventions within the local context. Periodic clinical audits are required to inform and update health care protocols. This study aimed to review morbidity and mortality in very low birth weight (VLBW) infants in 2013 compared with similar data from 2006/2007.

**Methods:**

We performed a retrospective review of patients’ records from a neonatal computer database for 562 VLBW infants. These neonates weighed between 500 and 1500 g at birth, and were admitted within 48 hours after birth between 01 January 2013 and 31 December 2013. Patients’ characteristics, complications of prematurity, and therapeutic interventions were compared with 2006/2007 data. Univariate analysis and multiple logistic regression were performed to establish significant associations of various factors with survival to discharge for 2013.

**Results:**

Survival in 2013 was similar to that in 2006/2007 (73.4% vs 70.2%, p = 0.27). However, survival in neonates who weighed 750–900 g significantly improved from 20.4% in 2006/2007 to 52.4% in 2013 (p = 0.001). The use of nasal continuous positive airway pressure (NCPAP) increased from 20.3% to 62.9% and surfactant use increased from 19.2% to 65.5% between the two time periods (both p < 0.001). Antenatal care attendance improved from 54.4% to 70.6% (p = 0.001) and late onset sepsis (>72 hours after birth) increased from 12.5% to 19% (p = 0.006) between the two time periods. Other variables remained unchanged between 2006/2007 and 2013. The main determinants of survival to discharge in 2013 were birth weight (odds ratio 1.005, 95% confidence interval 1.003–1.0007, resuscitation at birth (2.673, 1.375–5.197), NCPAP (0.247, 0.109–0.560), necrotising enterocolitis (4.555, 1.659–12.51), and mode of delivery, including normal vaginal delivery (0.456, 0.231–0.903) and vaginal breech (0.069, 0.013–0.364).

**Conclusions:**

There was a marked improvement in the survival of neonates weighing between 750 and 900 g at birth, most likely due to provision of surfactant and NCPAP. Provision of NCPAP, prevention of necrotising enterocolitis, and control of infection need to be prioritised in VLBW infants to improve their outcome.

## Background

In 2013, neonatal mortality accounted for almost 45% of deaths in children younger than 5 years of age [1]. Only 27 of 138 developing countries are likely to achieve the fourth Millennium Development Goal of reducing under-5 mortality by two thirds before 2015, and South Africa is not among these countries [2]. The neonatal mortality rate in South Africa is lower than the global average but is approximately five-fold that in some European and Scandinavian countries [2]. Preterm birth is the most important cause of neonatal mortality [3]. In 2013, complications relating to prematurity were the second largest cause of death in children younger than 5 years old, with infectious causes accounting for approximately half of these deaths [1]. Most neonatal deaths occur within the first week of life and two thirds could be prevented by provision of adequate health care [1].

To achieve the Every Newborn target of less than 10 neonatal deaths per 1000 births by 2035, the most important causes of neonatal death need to be determined [4]. Very low birth weight (VLBW) neonates (birth weight <1500 g) comprise a high-risk group with considerable mortality. Survival of VLBW infants in South Africa is reported to be just over 70% [5,6]. Although this survival rate is better than some other African countries, it is far worse than other developing countries [7,8]. Efforts to reduce neonatal mortality, and especially that of VLBW infants, must become a health priority in South Africa.

New developments in neonatal care are associated with improved survival in preterm infants. These include *in utero* transfer, antenatal steroids, early surfactant therapy and extubation to nasal continuous positive airway pressure (NCPAP) [9], promotion of breastfeeding, and kangaroo mother care (KMC). Neonatal networks, such as the Vermont Oxford Network (VON) [10], have been developed to improve the standard and safety of neonatal care through clinical audits and quality control. South Africa is a middle-income country with limited health resources, resulting in rationing of care to extremely low birth weight infants, many of whom are not offered mechanical ventilation. Newborns who weigh <900 g are not routinely provided with intermittent positive pressure ventilation (IPPV) in our institution, based on anticipated poor outcome, prolonged ventilation, and high use of resources. However, less costly and invasive measures, such as INSURE (Intubate-SURfactant-Extubate) and NCPAP have been shown to significantly improve survival in this group of neonates [11].

Ongoing clinical audits, incorporating regional neonatal and obstetric services within a perinatal network, provide current, local statistics that can be used to inform and monitor interventions to improve neonatal outcome. The Perinatal Problem Identification Programme [12] is a South African national audit of neonatal deaths, which considers the obstetric and neonatal causes of death, as well as avoidable factors. Additional information on other outcomes and interventions is beneficial. Therefore, an integrated approach, incorporating regional neonatal and obstetric services, would be helpful in developing integrated perinatal care programmes. Although the VON [10] considers this information, certain data, such as maternal human immunodeficiency virus (HIV) and syphilis status, are lacking, which are of great local importance. Therefore, a locally relevant system of clinical audits needs to be developed.

Health care protocols need to be regularly reviewed and modified to accommodate new therapies and address specific issues in a particular setting. This is especially relevant in low- and middle-income countries with limited health resources, insufficient equipment, and a lack of adequately skilled staff. Interventions shown to improve outcomes in a high-income setting may not be as effective in low- and middle-income countries. There is limited information regarding the care and outcome of VLBW infants in sub-Saharan Africa. A study conducted in the Charlotte Maxeke Johannesburg Academic Hospital (CMJAH) neonatal unit in 2006/2007 showed a survival rate of 70.5% with birth weight, NCPAP, necrotising enterocolitis (NEC), hypotension, sex, and place of birth as significant predictors of survival [13]. NCPAP was introduced in the unit in June 2006 and was initially only provided to a few neonates weighing >900 g. Neonates with a birth weight <900 g were not provided with ventilatory support or surfactants at the time. INSURE and NCPAP have gradually become first-line therapies for all VLBW babies >750 g at birth with hyaline membrane disease (HMD) in the CMJAH neonatal unit. The 2006/2007 study also identified other areas for improvement, such as attendance at antenatal care and the use of antenatal steroids.

The present study aimed to review the morbidity and mortality of VLBW infants at the CMJAH in 2013 and to compare these data with data from 2006/2007 in the same unit. The acquired information would be useful for revising and updating health care protocols to improve outcomes in this group of neonates in sub-Saharan Africa.

## Methods

This study was a retrospective review of patients’ records that were obtained from the CMJAH neonatal computer database, which is kept for clinical audit. All neonates who weighed between 500 and 1500 g, who were born between 01 January 2013 and 31 December 2013, and who were admitted to the CMJAH neonatal unit within 48 hours of birth, were eligible for inclusion. The primary outcome was survival to discharge from hospital. Secondary outcomes were rates of complications and therapeutic interventions and risk factors for mortality.

A similar study was conducted in the CMJAH unit from 1 July 2006 to 30 June 2007 [13]. The definition used for VLBW infants at that time considered all neonates weighing 1500 g or less and neonates were enrolled within 24 hours of birth. The current study used the definition of VLBW infants as defined by the VON [12] as neonates with a birth weight between 500 and 1500 g. To have a valid group for comparison, neonates with the same inclusion criteria as the current study were extracted from the initial data set (i.e., those who were admitted to the neonatal unit within 48 hours of birth between 1 July 2006 and 30 June 2007 with a birth weight of 500–1500 g). Therefore, this dataset was slightly different from that in the previously published article [13] and was used as the baseline in the current comparison.

### Hospital facilities and services

The neonatal unit at the CMJAH has a six-bed labour ward nursery, where newborns are initially admitted and stabilised. There is a 35-bed high-care nursery where intravenous fluids, antibiotics, NCPAP, surfactant therapy, supplemental oxygen, and phototherapy are provided. Additionally, this hospital also has a 20-bed ward where neonates await weight gain for discharge and a 15-bed kangaroo mother care (KMC) ward. IPPV is provided in a shared 14-bed paediatric/neonatal intensive care unit. High-frequency ventilation, paediatric cardiology, paediatric neurology, and paediatric surgery are available within the facility. High-flow oxygen therapy was not used in the unit at the time. The neonatal and obstetric services are referral centres for the surrounding midwife obstetric units and district hospitals. Neonates whose current weight was above 1000 g were sent to KMC just prior to discharge when they were clinically stable, off supplemental oxygen, and tolerating full enteral feeds. Whenever possible, these neonates were transferred out to KMC facilities in regional hospitals. Neonates were discharged home from the CMJAH once they had achieved a current weight of 1600 g, were taking full feeds from either the breast or cup, and maintaining body temperature and blood glucose levels. Neonates were occasionally discharged home on supplemental oxygen.

### Neonatal care

Neonates were managed according to standard unit protocols by attending neonatologists and paediatric registrars. All neonates, irrespective of birth weight, received warmth, supplemental oxygen via low-flow nasal cannulae, intravenous fluids, phototherapy, and antibiotic therapy as required. Cranial ultrasound was performed on all VLBW infants within the first week of life. Screening for retinopathy of prematurity (ROP) was performed by an ophthalmologist at the later age of either 4 to 6 weeks of chronological age or at 31 to 33 weeks’ corrected gestational age. Packed red cell transfusions were provided if the neonate showed symptoms of anaemia with a haemoglobin (Hb) level below 8 g%, at an Hb level below 10 g% if the neonate was receiving supplemental oxygen, or at an Hb level below 12 g% if the neonate was receiving IPPV.

Because of limited resources, the neonatal unit has a policy of rationing ventilatory support according to birth weight [13]. Rescue surfactant therapy that was administered as INSURE and NCPAP was provided as a first-line therapy in all VLBW infants >750 g at birth with HMD who showed signs of respiratory failure. Ventilation (IPPV) was provided to those infants >900 g who showed evidence of respiratory failure on NCPAP or became apnoeic. Newborns who were below the weight cut-off were occasionally provided with support at the discretion of the attending medical staff.

### Neonatal database and ethical approval

Detailed information, including birth factors, therapeutic interventions, complications of prematurity, and clinical outcome, was collected upon discharge from hospital for each patient. This information was entered into a neonatal database that was maintained for the purpose of clinical audits and quality control. This database was managed using Research Electronic Data Capture tools hosted at the University of the Witwatersrand [14]. Data for each eligible VLBW patient were obtained from the computer database and analysed. Each case was de-identified for the purpose of confidentiality. Ethical approval for the study was obtained from the Human Research Ethics Committee of the University of the Witwatersrand.

### Definitions

Standard definitions as per the VON [10] were used. NEC was considered as modified Bell’s stage 2 or 3 [15] and peri-intraventricular haemorrhage (IVH) was classified using Papile’s staging [16]. At the time of the study, the neonatal unit of the CMJAH did not submit data to the VON. Maternal HIV referred to mothers who were HIV positive and not necessarily those on anti-retroviral treatment or those who had AIDS. Chorioamnionitis was defined as premature and/or prolonged rupture of the membranes, fever, and foul-smelling liquor in mothers. Neonates were considered to be small for gestational age if the birth weight was below the 10^th^ percentile on the Fenton growth charts [17]. Resuscitation at birth was defined as the need for bag mask ventilation, chest compressions, or intubation and ventilation. Severe IVH was considered to be either grade 3 or 4. Sepsis was classified as culture-proven bacterial or fungal sepsis only; suspected sepsis or clinical sepsis was classified as no sepsis. Birth defects were defined as life-threatening anomalies at birth. Respiratory failure was defined as oxygen saturation below 88% in 60% supplemental oxygen, respiratory acidosis (pH <7.25 with PaCO_2_ > 60 mmHg), or clinical marked respiratory distress. Ventilatory support referred to the most invasive level each neonate received (i.e., those who were treated with both NCPAP and IPPV were classified in the IPPV group). Neonates who were transferred out to regional hospitals for KMC were classified as survivors.

### Statistical analysis

Statistical analysis was performed using SPSS (IBM Corp released 2013 IBM SPSS Statistics for Windows Version 22.0, Armonk NY). Data are described using standard statistical methods. Frequency tables and percentages with 95% confidence intervals were used for categorical variables. Continuous variables (normally distributed) are summarised using mean and standard deviation (SD). Results are reported as mean (±SD). Obstetric and labour room information is reported per neonate (not per mother) to allow for multiple pregnancies and to concur with the aim of the study. Univariate analysis was performed using cross tabulations with the chi square test to compare categorical variables. Continuous variables were normally distributed and thus compared using unpaired t-tests. A p value less than 0.05 was considered significant. Various demographic and birth factors, complications of prematurity, and therapeutic interventions were compared between 2006/2007 and 2013. Ventilatory support was different among the various birth weight categories. Therefore, neonates were stratified into weight groups as follows: <750 g, no ventilatory support (no IPPV or NCPAP); 750–900 g, NCPAP only if required; 900–1500 g, NCPAP and IPPV if required. These weight groups were then compared for IPPV, surfactant use, NCPAP, and survival.

Further analysis was performed considering the 2013 data only. Univariate analysis was performed to determine significant associations of various factors with survival at discharge. A multiple logistic regression model with survival as the binary outcome variable, using a forward entry conditional model, was then performed. Variables that were significantly associated with survival in univariate analysis were included in the model. Logistic regression was repeated excluding neonates with less than 750 g birth weight to control for possible selection bias because these neonates did not receive ventilatory support.

## Results

The sample comprised 562 VLBW infants, including 20 who died in the delivery room. The mean birth weight was 1120.0 (±248) g in 2013 and 1127.0 (±233) g in 2006/2007, with no difference between the two time periods (p = 0.64). Mean gestational age was significantly lower in 2013 than in 2006/2007 (29.3 [±2.8] vs 29.9 [±2.9] weeks, p < .001). The mean duration of hospital stay was 28.2 (±21.8) days in 2013 and 25.8 (±22.1) days in 2006/2007, with no difference between the two time periods (p = 0.08). Thirty-two (5.6%) neonates were transferred to regional step-down facilities. The mean birth weight of these neonates was 1166.6 (±195.7) g (p = 0.92 compared with the main sample) and the mean gestational age was 29.9 (±2.16) weeks (p = 0.98 compared with the main sample).

### Population characteristics

#### Birth factors

Demographic and birth characteristics are shown in Table 1. The mean maternal age was 28.1 (±6.1) years. Fifteen (2.7%) mothers were teenagers and 165 (29.4%) were primiparous. Attendance at antenatal care significantly improved from 54.4% to 70.6% (p = 0.001) and the number of neonates who were born SGA decreased from 37.6% to 30.9% in 2006/2007 to 2013 (p = 0.038).

**Table 1:** **Demographic and birth characteristics of VLBW infants who were admitted to the CMJAH**

**Variable**	**2013 (total 562)**	**2006/2007 (total 463)**	**P Value**
	**Number (%) [95%CI]**	**Number (%)[95% CI]**	
**Multiple gestation**	90 (16) [13.2- 19.3]	77 (16.6) [12.7 – 21.3]	0.85
**Maternal HIV**	160 (28.5)[24.9 – 32.3]	119 (25.7)[21.6 – 29.8]	0.26
**Maternal syphilis**	6 (1.1) [0.4 – 2.3]	11 (2.4) [1.1 – 3.9]	0.18
**Antenatal steroids**	220 (39.1)[35.2 – 43.3]	172 (37.1)[32.6 – 41.5]	0.4
**Antenatal care**	397 (70.6)[66.7 – 74.3]	252 (54.4)[49.4 – 59]	0.001
**Chorioamnionitis**	14 (2.5) [1.6 – 4.1]		
**Maternal hypertension**	130 (23.1)[19.8 – 26.8]		
**Inborn**	472 (84)[80.7 – 86.8]	378 (81.6) [78.7 – 85.7]	0.49
**Born outside a health facility**	33 (5.9) [4.2 – 8.1]	34 (7.3) [5.0 – 9.6]	0.41
**C-section**	311 (55.4) [51.2 – 59.4]	238 (51.4) [46.1 – 58.5]	0.23
**SGA**	174 (30.9))[27.3 – 34.9]	174 (37.6) [33.3-42.1]	0.03
**Resuscitation at birth**	203 (36.1)[32.2 – 40.3]	152 (32.8) [28.5 – 37.1]	0.31
**Female**	308 (54.8)[50.7 – 58.9]	238 (53.2)[48.3 – 57.7]	0.37

### Complications of prematurity and therapeutic interventions

**Figure 1:**
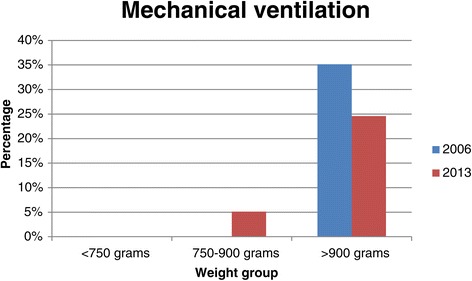
**Mechanical ventilation by birth weight category in VLBW infants with HMD at two time periods.** Significantly fewer babies who weighed > 900 g were ventilated in 2013 compared to 2006/2007 (p = 0.008).

**Figure 2:**
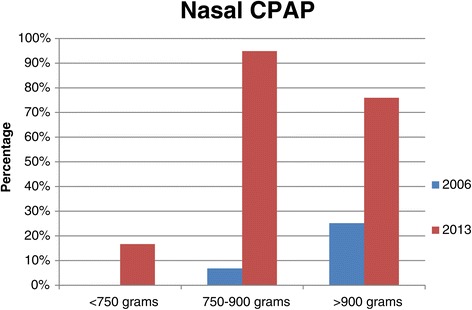
**NCPAP use in VLBW infants with HMD by birth weight at two time periods.** There was a significant increase in NCPAP use between 2006/2007 and 2013 for the weight categories of 750–900 g and >900 g (both p < 0.001).

**Figure 3:**
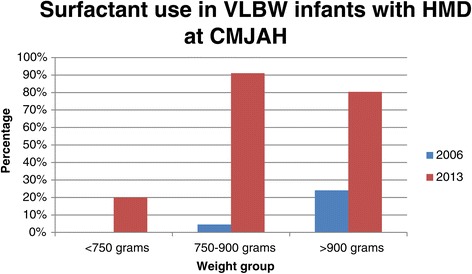
**Surfactant use in VLBW infants with HMD by birth weight category between two time periods.** There was a significant increase in surfactant use for all weight categories (<750 g, p = 0.024; 750–900 g, p < 0.001; >900 g, p < 0.001).

There was a significant increase in the number of neonates with HMD from 63.7% in 2006/2007 to 83.6% in 2013 (p < 0.001, Table 2). Figure 1 shows IPPV by birth weight for neonates with HMD. Significantly fewer neonates with HMD received IPPV in 2013 (20.9%) than in 2006/2007 (32.9%). Figure 2 shows NCPAP use by birth weight in neonates with HMD and Figure 3 shows surfactant use in neonates with HMD by birth weight. There was a significant increase in NCPAP use for neonates >750 g and in surfactant use in all weight categories between 2006/2007 and 2013. There was a marked increase in the use of NCPAP and surfactant between 2006/2007 and 2013. Significantly fewer neonates with HMD received NCPAP and were provided with surfactant in 2006/2007 than in 2013 (both p < 0.001). Most neonates who were treated with NCPAP (94.6%; 335/354) received surfactant therapy. There was also a significant increase in late onset sepsis from 12.5% in 2006/2007to 19% in 2013 (p = 0.006). One neonate was discharged home on oxygen.

**Table 2:** **Complications of prematurity and therapeutic intervention for VLBW infants who were admitted to the CMJAH**

	**2013 (Total 562)**	**2006/2007 (Total 463)**	**P Value**
**Variable**	**Number (%) [95%CI]**	**Number (%)[95%CI]**	
**HMD**	470 (83.6)[80.3 – 86.5]	295 (63.7) [59.2 – 68]	<0.001
**Surfactant**	368 /470 (78.3)[74.4 – 81.8]	89/295 (30.2)[25.2-35.6]	<0.001
**IPPV**	98/470 (20.9) [17.4 – 24.8]	97/295 (32.9) [27.8 – 38.4]	<0.001
**NCPAP**	354/470 (75.3) [71.2 – 79.0]	94 /470(31.9) [26.8 – 37.4]	<0.001
**PDA**	54 (9.6) [7.4-12.3]	26 (5.6) [3.9 – 8.1]	0.02
**Ligation of Patent Ductus Arteriosus (PDA)**	3 (0.5) [0.18- 1.5]	-	
**Blood Transfusion**	146 (26) [22.8 – 29.8]	106 (22.9) [19.2 – 26.6]	0.26
**Late onset sepsis**	107 (19) [16 – 22.5]	58 (12.5) [9.1 – 16.6]	0.01
**NEC**	41 (7.3) [5.4 – 9.8]	26 (5.6) [3.7 – 7.8]	0.34
**NEC surgery**	10 (1.8) [0.97 – 3.3]	-	
**Other surgery**	11 (2.0) [1.1 – 3.4]	-	
**Pneumothorax**	4 (0.7) [0.28 – 1.8]		
**Severe IVH**	22/297 (7.4)^*^[4.9 – 11]	20 / 321(6.0)^*^ [3.9 – 9.1]	0.89
**Cystic periventricular leukomalacia (PVL)**	2 /297(0.6)^*^ [0.12 – 2.7]	4 /321(1.2)^*^ [0.3 – 2.5]	0.52
**Severe ROP**	3/144 (2.1)^*^ [0.71 – 5.9]		
**Discharged on human milk feeds**	126/413 (30.5)^**^ [26.3 – 35.1]		

*percentage of babies screened.

**percentage of those discharged.

Not all neonates were screened for IVH or ROP. Cranial ultrasound was performed in 52.8% (297/562) of patients and only 25.6% (144/562) were screened for ROP. Many neonates did not have a cranial ultrasound for a number of reasons. Some of them died early before ultrasound could be performed, some were well and were sent to KMC early before having ultrasound, and shortages of staff and equipment sometimes resulted in cranial ultrasound being unavailable. Many neonates did not have screening for ROP because they were discharged prior to the required age for screening, which was then performed on an outpatient basis.

### Mortality

**Figure 4:**
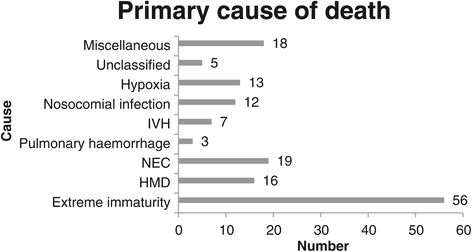
**Primary cause of death in VLBW infants at the CMAJH in 2013.**

**Figure 5:**
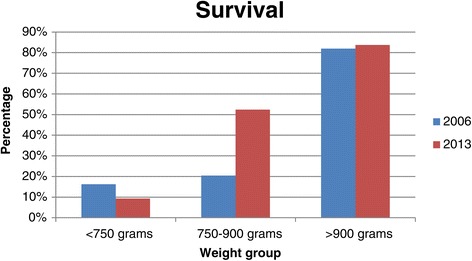
**Survival of VLBW infants by birth weight category at two time periods.** Survival of VLBW infants who weighed 750–900 g at birth significantly improved between 2006/2007 and 2013 (p = 0.001).

The overall survival rate was 413/562 (73.4%; 95% confidence interval 69.6–77%). The primary cause of death is shown in Figure 4. The most common cause of death was extreme multi-organ immaturity in 56/149 (37.5%) patients. The survival of VLBW infants in 2013 was not different from that in 2006/2007 (325/463 [70.2%]; 95% confidence interval 65.9–74.2; p = 0.27). However, survival of neonates weighing 750–900 g significantly improved from 20.4% in 2006/2007 to 52.4% in 2013 (p = 0.001), while survival in the other birth weight categories remained unchanged (Figure 5). The duration of hospitalisation was significantly longer in survivors than in non-survivors (35.52 [19.89] days vs 7.77 [12.57] days, p = 0.001). The majority of deaths (111/149, 74.49%) occurred in the early neonatal period. There were 20 delivery room deaths. These neonates had a mean birth weight of 870.8 (271) g and a gestational age of 26.7 (3.6) weeks. The cause of death was extreme multi-organ immaturity in 11 (55%), hypoxia in four (20%), HMD in three (15%), and congenital abnormalities in two (10%) neonates.

#### Univariate analysis of predictors of survival for 2013

Birth weight was significantly lower in non-survivors (911.4 [227] g) than in survivors (1191.3 [212] g, p < 0.001). Similarly, gestational age was significantly lower in non-survivors (27.4 [2.5] weeks) than in survivors (29.9 [2.6] weeks, p < 0.001). Temperature at admission was significantly lower in non-survivors than in survivors (35.1 [1.7] °C vs 35.8 [1.2] °C, p < 0.001). The neonate’s sex was not associated with outcome. Other significant predictors of survival at discharge are shown in Tables 3 and 4. Place of birth, antenatal care, antenatal steroids, maternal hypertension, maternal HIV infection, mode of delivery, resuscitation at birth, and major birth defects were significantly associated with outcome (Table 3). IPPV, NCPAP, surfactant therapy, patent ductus arteriosus, and NEC were significantly associated with outcome (Table 4). Fifty-one percent of neonates received KMC, and KMC was strongly associated with survival. Five of the 237 babies (1.7%) who had KMC died as compared to 118 of the 237 babies (49.8%) who did not have KMC (p < 0.001). However, there was marked selection bias because neonates in this study were sent to KMC just prior to discharge. Therefore, KMC was not included in the multivariate analysis. Major birth defects were predictive of survival in univariate analysis, but were also excluded from multivariate analysis because only life-threatening defects were considered. Other factors, including the duration of ventilation, the duration of nasal CPAP, the age of receiving surfactants, hypoglycaemia, atelectasis, pulmonary haemorrhage, pneumothorax, blood transfusion, exchange transfusion, and sepsis (both early and late onset), were not significant predictors of mortality.

**Table 3:** **Obstetric and birth-related factors associated with survival to discharge for VLBW infants in 2013**

**Variable (number)**	**Died/survived**	**Mortality percentage [95% CI]**	**P value**
**Birthplace**			
**Inborn (472)**	121/351	25.6 [21.9 – 29.7]	
**Born Outside Health facility (33)**	17 /16	51.5 [35.2 – 67.5]	0.006
**Born at another hospital (36)**	6 /30	16.6 [7.9 – 31.9]	
**Born at Midwife obstetric unit (19)**	5/14	26.3 [11.8 – 48.8]	
**Antenatal care**			
**Yes (397)**	92/305	23.1 [19.3 – 27.6]	0.016
**No (140)**	47 /93	33.5 [26.3 – 41.7]	
**Antenatal steroids**			
**Yes (220)**	44 /176	20 [15.3 – 25.8]	0.001
**No (288)**	95/193	32.9 [27.8 – 38.6]	
**Maternal hypertension**			
**Yes (126)**	24 /106	18.4 [12.7 – 26]	0.022
**No (366)**	105 /261	28.6 [24.3 – 33.5]	
**Maternal HIV**			
**Yes (160)**	47 /113	29.3[22.9 – 36.9	0.038
**No (362)**	76 /286	20.9[17.1 – 25.5]	
**Mode of delivery**			
**NVD (221)**	87/134	39.3[33.2 – 45.6]	
**Vaginal breech (18)**	9 /9	50 [29 – 71]	
**CS elective (15)**	3 /12	20 [7 – 45.2]	<0.001
**CS emergency (296)**	48/248	16.2 [12.5 – 21]	
**SGA**			
**Yes (174)**	30/144	17.2 [12.4 – 23.5]	
**No (384)**	112/272	29.1 [24.8-33.9]	<0.01
**Resuscitation at birth**			
**Yes (203)**	93/110	45.8 [39.1 – 52.7]	<0.001
**No (344)**	53/291	21.7 [17 – 27.3]	
**Major birth defect**			
**Yes (12)**	7/5	58.3 [31.9 – 80.7]	0.013
**No (536)**	140 / 396	26.1 [22.6 – 30]

**Table 4:** **Complications of prematurity and therapeutic interventions significantly associated with survival in VLBW infants in 2013**

**Variable**	**Died/survived**	**Percentage mortality**	**P value**
**IPPV**			
**Yes (98)**	38 /60	38.8 [29.7 – 48.7]	
**No (415)**	89 /329	21.3 [17.6 – 25.5]	<0.001
**Nasal CPAP**			
**Yes (290)**	61/229	21 [16.7 – 26.1]	
**No (61)**	25 /36	40.9 [29.5 – 53.5]	0.001
**Surfactant therapy**			
**Yes (368)**	87 /281	23.6 [19.6 -28.2]	
**No (189)**	62/127	32.8 [26.5 – 36.8]	0.021
**PDA**			
**Yes (54)**	7 /47	32.8 [26.5 – 39.8]	
**No (482)**	121 /361	25.1 [21.4 – 29.2]	0.047
**NEC**			
**Yes (41)**	18 /23	43.9 [29.9 – 59]	
**No (495**	111 /384	22.4 [19 – 26.3]	0.002

#### Multiple regression analysis

Results of multiple regression analysis for the whole group are shown in Table 5. Significant predictors of mortality were birth weight, resuscitation at birth, NCPAP, NEC, and mode of delivery. Multiple logistic regression excluding neonates <750 g at birth (no NCPAP or IPPV) showed that significant predictors of mortality were birth weight (p < 0.001), maternal HIV status (p = 0.024), resuscitation at birth (p = 0.002), NCPAP (p = 0.028), and NEC (p = 0.002).

**Table 5:** **Multivariable logistic regression for factors associated with survival to discharge for VLBW infants in 2013**

**Variable**	**Odds ratio (95%CI)**	**Significance**
Birthweight	1.005 (1.003 – 1.007)	<0.001
Mode of delivery		
-NVD	0.456 (0.231 - .0.903)	0.02
-Vaginal breech	0.069 (0.013 – 0.364)	0.002
Resuscitation at birth	2.673 (1.375 – 5.197)	0.004
NCPAP	0.247 (0.109 – 0.560)	0.001
NEC	4.555 (1.659 – 12.510)	0.003

## Discussion

The number of VLBW infants is increasing, with an increase of 18.5% of VLBW admissions to the CMJAH in 2013 compared with 2006/2007. Care of these infants should become a health priority in the coming decade. Regular review of outcomes and adjustment of health protocols are essential to ensure the best possible outcome of patients. There was a marginal improvement in the overall survival of VLBW infants in this unit, from 70.2% in 2006/2007 to 73.4% in 2013, but this did not reach statistical significance. These survival rates are similar to those in Korea (6), but remain below those of high-income countries (14, 15). Different interventions are provided for different birth weights in the CMJAH neonatal unit. In the current study, stratifying for birth weight showed that survival of VLBW infants weighing 750–900 g significantly improved from 20.4% in 2006/2007 to 52.4% in 2013, whereas that for infants <750 g and those who weighed 900–1500 g did not change. The reason for this finding can be attributed to the introduction of NCPAP and INSURE for the group of neonates weighing 750–900 g, whereas the management of neonates in the other weight groups remained unchanged between the two time periods. During 2006/2007, only 6.8% of neonates with HMD who weighed 750–900 g at birth were offered NCPAP compared with 94.8% in 2013. The number of neonates who weighed >900 g with HMD requiring IPPV significantly decreased from 35.1% in 2006/2007 to 24.6% in 2013. Importantly, NCPAP is a cheaper therapeutic option than mechanical ventilation with its attendant difficulties and complications. Based on this markedly improved survival of ELBW infants, perhaps physicians should review the birth weight cut-off for NCPAP and INSURE, and offer this to all neonates with RDS, regardless of their birth weight. Provision of available therapeutic interventions to every neonate, irrespective of birth weight, is a major factor in better survival rates of VLBW infants in high-income compared with low- and middle-income countries [18].

The most significant predictor of survival in the present study in 2013 was birth weight, which is consistent with previous findings in 2006/2007. Other significant predictors of survival in the present study were NCPAP, NEC, resuscitation at birth, maternal HIV status, and mode of delivery. These findings are slightly different to those in 2006/2007 where hypotension, sex, and birth outside a health facility were associated with outcome, but these factors were not associated in the 2013 study. However, NCPAP and NEC remained significantly associated with outcome in both time periods. Other significant differences between the two time periods were an increase in attendance at antenatal care, HMD, and late onset sepsis, with a decrease in neonates who were SGA. The significant increase in the number of neonates with HMD between the two time periods was unexpected, but may be associated with the decrease in the number of neonates who were SGA [19]. Despite this increase in HMD, the survival rate increased over time (as discussed above).

Some authors have suggested that there is little additional benefit in using other predictors of outcome over birth weight in resource-constrained settings [20]. However, both studies at the CMJAH showed other modifiable factors to be significantly associated with survival, with particular emphasis on NEC and NCPAP. Survival of VLBW infants in the local context can be improved with low technology and relatively inexpensive interventions (e.g., NCPAP) in a regional hospital setting, provided there is adequate equipment and properly trained staff. Health protocols at the CMJAH should be developed to include promotion of breastfeeding, adequate neonatal resuscitation, prevention of mother-to-child transmission of HIV, and provision of NCPAP. Notably, administration of antenatal steroids [21] and prevention of hypothermia [22] should also be addressed, even though these factors were not significant in multiple regression analysis. The rate of late onset sepsis (LOS) significantly increased from 12.5% in 2006/2007 to 19% in 2013. This finding might be due to overcrowding and overuse of broad spectrum antibiotics. Surprisingly, LOS was not a significant cause of mortality in the current study. This finding may be due to a strong association between NEC and nosocomial infection because 42% of neonates who died of NEC had nosocomial infection. Although LOS was not a risk for mortality, control of infection should also be prioritised because LOS is associated with increased costs of care, prolonged hospitalisation, and an adverse outcome [23]. Although vaginal delivery is associated with a poor outcome, it may not be feasible to deliver all preterm infants by caesarean section, particularly in a resource-constrained setting.

In the current study, notably, several factors did not improve over time. Administration of antenatal steroids remained low. Many mothers present late in labour and do not attend antenatal care. Therefore, there may be limited opportunities for obstetricians to administer steroids [24]. Maternal HIV infection remains a major problem, affecting almost one third of mothers.

Certain complications of prematurity in the current study were low, including pneumothorax, cystic periventricular leukomalacia, severe peri-intraventricular haemorrhage P-IVH, and ROP. This may reflect the relatively poor survival of ELBW infants, but many of the neonates in the present report were not screened. Therefore, these figures may be under-reported. Reliable information on long-term outcome (not only survival) is essential. Therefore, protocols must be put in place to ensure that all neonates are screened for ROP and have cranial ultrasound performed. The presence of ROP, IVH, and periventricular leukomalacia can be used as proxy indicators of a long-term poor outcome.

## Conclusion

The mortality rate of VLBW infants did not significantly change between 2006/2007 and 2013. The greatest improvement in survival was observed in VLBW infants who weighed 750–900 g at birth. This most likely reflects the provision of surfactants and NCPAP to this weight category of infants. Health protocols, including simple, inexpensive interventions (e.g., adequate neonatal resuscitation, NCPAP, prevention of mother-to-child transmission of HIV, and measures to prevent NEC), should improve survival to discharge in VLBW infants. Although LOS is not associated with mortality, there is an alarming increase in LOS, and infection control measures must also be prioritised. These interventions will not only improve the survival of VLBW infants but also result in lower costs in the care of these neonates.

## Abbreviations

CS: Caesarean section
CMJAH: Charlotte Maxeke Johannesburg Academic Hospital
ELBW: Extremely low birth weight
Hb: Haemoglobin
HIV: Human immunodeficiency virus
HMD: Hyaline membrane disease
INSURE: Intubate-SURfactant-Extubate
IPPV: Intermittent positive pressure ventilation
IVH: Intraventricular haemorrhage
KMC: Kangaroo mother care
LOS: Late onset sepsis
NCPAP: Nasal continuous positive airways pressure
NEC: Necrotising enterocolitis
PDA: Patent ductus arteriosus
PVL: Periventricular leukomalacia
ROP: Retinopathy of prematurity
SGA: Small for gestational age
VLBW: Very low birth weight
VON: Vermont Oxford Network
